# Reconstructing birth in *Australopithecus sediba*

**DOI:** 10.1371/journal.pone.0221871

**Published:** 2019-09-18

**Authors:** Natalie M. Laudicina, Frankee Rodriguez, Jeremy M. DeSilva

**Affiliations:** 1 Department of Anthropology, Boston University, Boston, Massachusetts, United States of America; 2 Biomedical Sciences Department, Grand Valley State University, Grand Rapids, Michigan, United States of America; 3 Department of Anthropology, Dartmouth College, Hanover, New Hampshire, United States of America; 4 Evolutionary Studies Institute, University of the Witwatersrand, Johannesburg, South Africa; University of Delaware, UNITED STATES

## Abstract

Hominin birth mechanics have been examined and debated from limited and often fragmentary fossil pelvic material. Some have proposed that birth in the early hominin genus *Australopithecus* was relatively easy and ape-like, while others have argued for a more complex, human-like birth mechanism in australopiths. Still others have hypothesized a unique birth mechanism, with no known modern equivalent. Preliminary work on the pelvis of the recently discovered 1.98 million-year-old hominin *Australopithecus sediba* found it to possess a unique combination of *Homo* and *Australopithecus*-like features. Here, we create a composite pelvis of *Australopithecus sediba* to reconstruct the birth process in this early hominin. Consistent with other hominin species, including modern humans, the fetus would enter the pelvic inlet in a transverse direction. However, unlike in modern humans, the fetus would not need additional rotations to traverse the birth canal. Further fetal rotation is unnecessary even with a *Homo*-like pelvic midplane expansion, not seen in earlier hominin species. With a birth canal shape more closely associated with specimens from the genus *Homo* and a lack of cephalopelvic or shoulder constraints, we therefore find evidence to support the hypothesis that the pelvic morphology of *Australopithecus sediba* is a result of locomotor, rather than strictly obstetric constraints.

## Introduction

Among primates, the mechanics of human birth are thought to be unique, typically involving a vertex presentation of the fetal head and a multi-rotational pattern of the neonate through the birth canal. The evolutionary explanation for the difficulty of human birth, termed the “obstetrical dilemma” [1], posits that a large, encephalized infant combined with pelvic modifications adapted for bipedality result in an exceeding difficult parturition in humans. In particular, compared to the ape pelvis, the human pelvis is anteroposteriorly (AP) shorter and mediolaterally (ML) broader, which effectively constricts dimensions of the birth canal relative to the modern ape condition.

Humans have adapted to the obstetrical dilemma in part by recruiting attendants who aid the mother in giving birth [2, 3]. Although recent research has found that the obstetrical dilemma, as originally conceived, fails to explain the timing of human birth [4–8], it is generally accepted that humans experience more difficult births than do our closest living relatives, the great apes, who typically labor for less time and without the assistance of others [2, 9–12]. Non-human apes achieve this relative ease of parturition through a smaller neonatal size (cranium and body), a more spacious birth canal, and a consistent orientation of the neonate’s head throughout the birth canal [2, 12]. Although variable [9, 13], the majority of ape neonates present in the occiput-posterior position.

Thus, the birth process in non-human apes is relatively simple compared to humans. The birth canal and the neonate’s head are elongated in the same plane (anterior-posterior) and the birth canal does not change shape or dimensions [2]. In contrast, the modern human pelvis is shaped in a manner that typically requires fetal rotation, both of the cranium and shoulders [2, 14, 15]. As a human neonate descends into the pelvic inlet, its cranium is aligned obliquely, or transversely, in the birth canal due to the shortened anterior-posterior dimension of the maternal pelvic inlet [2]. Typically, the neonate's head will flex, tucking the chin to its chest, to achieve a shorter length along the suboccipito-bregmatic axis or plane, helping to alleviate the tight fit [2]. In contrast, the AP expanded pelvic inlet of non-human primates allows the neonate to align its head sagittally, without this initial rotation or need for neck flexion [2, 3, 14, 16, 17].

A further constraint is met in the midplane of the human maternal pelvis. The ischial spines constrict the transverse diameter and the anterior-posterior dimension becomes relatively elongated [2, 3, 14, 17]. Taking advantage of the maximum dimensions of the maternal pelvis [18], the neonate's head typically aligns the convex occiput against the complementary surface of the female pubis, resulting in an occiput-anterior birth position [2, 3, 14, 19]. In non-human primates, the occiput-posterior birth position allows the mother to assist the neonate out of the birth canal without risking injury. In humans, the baby is most commonly born occiput-anterior. In this position the mother may extend the neonatal neck, risking spinal injury if she tries to help it out herself [2, 3, 14]. Modern humans have overcome this predicament with birth assistants who aid the newborn safely out of the birth canal.

A final challenge that is too often ignored is imposed by the neonatal shoulders. Birth complications due to shoulder obstruction, or shoulder dystocia, occur in 1.4% of the U.S. population [20], of which 24.9% result in fetal injury [21]. Like the neonatal head, the broad, rigid shoulders follow the maximum dimensions of the maternal birth canal [2, 3, 14, 18]. After transverse descent into the inlet, the shoulders twist to be sagittally-aligned with the greatest axis of the maternal pelvis in the midplane. In exiting the birth canal, one shoulder will typically position under the pubic symphysis before the other shoulder, alleviating the tight fit [2, 3, 14].

When rotational birth evolved in hominins is unclear, partially due to the scarcity of female pelvic remains [2, 3, 14, 17, 22–24]. The platypelloid pelvis of the 3.18-million-year-old *Australopithecus afarensis* skeleton, A. L. 288–1, indicates that an *A*. *afarensis* neonate's head would probably have entered the pelvic inlet in a human-like transverse or oblique orientation [15–17, 23, 25, 26]. The Berge et al. [23] reconstruction suggest a more human-like neonatal flexion and rotation through the birth canal while the Tague and Lovejoy [17] reconstruction favors a continued transverse, or asynclitic, passage of the neonate throughout the birth canal. Incorporation of the neonatal shoulder dimensions suggest that a semi-rotational oblique birth would be most probable [15]. Another interpretation posits that this specimen is not a female, given the close fit between a hypothetical *A*. *afarensis* neonate and the A.L. 288–1 birth canal [25] (but see [27–29]). Although we do not agree with the conclusion that A.L. 288–1 is a male specimen, obstetric dimensions from both Tague and Lovejoy [17] and Häusler and Schmid [25] are utilized as a range.

Hypothesized birth mechanisms of *A*. *africanus* are similarly contentious based on varied pelvic reconstructions of Sts 14 [22, 23, 25, 30–32] and Sts 65 [33]. In A.L. 288–1, Sts 14, and Sts 65, a transverse entry into the pelvic inlet illustrates a beginning to the modern human birth mechanism [33], while midplane and outlet rotations remain debatable [2, 3, 14, 15, 17, 22–25].

The 0.9 to 1.4 Ma female *Homo erectus* pelvis, BSN49/P27, has a more gynecoid-shaped birth canal, distinct from the platypelloid pelvic shapes of the previously discussed australopiths [34] (but see [35]). The more expanded birth canal has been interpreted as an adaptation for birthing larger brained neonates in the genus *Homo*, although the mechanism of birth (i.e. rotational) was not explicitly discussed [34].

Reconstructions of the female Neandertal Tabun 1 pelvis show an expanded mediolateral dimension of the pelvic inlet and midplane combined with an expanded anterior-posterior outlet [36, 37]. These pelvic dimensions, as well as an increased neonatal cranial capacity in Neandertals, has led some researchers to infer a modern human-like rotational birth in this population [36, 38] while others have suggested a more primitive non-rotational transverse mechanism of delivery in their pelvic reconstruction [37]. Both interpretations are included in our comparison of these reconstructions to the *A*. *sediba* material.

This paper expands upon the female hominin pelvic sample to include *Australopithecus sediba* [39]. Dated to 1.977 million years old [40], the two partial skeletons of an adult female and juvenile male include pelvic material that combines australopith and early *Homo*-like anatomies in a small-brained species [41, 42]. Unlike the platypelloid pelves of *A*. *afarensis* and *A*. *africanus*, the *A*. *sediba* pelvis is more anterior-posteriorly expanded, like the pelves of *H*. *erectus* and modern humans, albeit to a lesser degree [42]. Kibii et al. [42] proposed that the presence of *Homo*-like features in a small-brained hominin implied that pelvic adaptations may be related to locomotion rather than to birth constraints. Testing this hypothesis necessitates a characterization of birth in *A*. *sediba*. Furthermore, even if pelvic changes are driven by locomotion, they may still impact the mechanism of delivery, as evidenced by the interplay between locomotion and parturition in early australopiths [17].

Here, we reconstruct the birth canal of the *A*. *sediba* female (MH2) and characterize the birth process based on a composite pelvis reconstruction and estimated neonatal cranial and shoulder dimensions.

## Materials and methods

Pelvic dimensions from female hominins *Australopithecus afarensis* (A.L. 288–1), *Australopithecus africanus* (Sts 14 and Sts 65), *Homo erectus* (BSN 49/P27), Neandertal (Tabun 1), modern humans (*Homo sapiens*), and chimpanzees (*Pan troglodytes*) were gathered [16, 17, 25, 30, 33, 34, 36, 37, 42–44] and compiled in Table 1.

**Table 1:** Female pelvic dimensions for fossil hominins, modern humans, and chimpanzees.

Specimen	Inlet–AP	Inlet–ML	Midplane–AP	Midplane—ML	Outlet–AP	Outlet—ML
A.L. 288-1^a^	76.0	132.0	72.0	101.0	71.0	96.0
A.L. 288-1^b^	79–81	123–126	112–115	86–89	90–92	83–86
Sts 14	83.0	116.8	73.3	93.1	NA	105.0
Sts 65	82.7–82.8	101.5–109.0	NA	NA	NA	NA
MH2	81.7	117.6	97.9	NA	97.4	NA
BSN 49/P27	98.0	124.5	111.5	114.5	*111.5	133.3
Tabun 1^c^	109–121	143–145	131–141	114–122	123–134	116–126
Tabun 1^d^	104	131	NA	NA	93	132
*H*. *sapiens*^e^	104.0 (n = 106)	134.0 (n = 119)	123.0 (n = 101)	106.0 (n = 18)	118.0 (n = 97)	122.0 (n = 70)
*H*. *sapiens*^f^ (n = 100)	91.0–112.0	123.0–135.0	112.9–138.0	NA	NA	111.8–127.0
*H*. *sapiens*^g^ (n = 6)	105.2	131.6	125.1	NA	119.4	NA
*Pan troglodytes*	136	90	117	71	122.4	105.0

Comparison of the pelvic dimensions reported for hominin and modern human female pelves [17, 25, 30, 33, 34, 36, 37].

A.L. 288-1^a^ measures are from Tague and Lovejoy [17].

The range reported for A.L. 288-1^b^ is from two versions of the Häusler and Schmid [25] A.L. 288–1 reconstruction. The sagittal midplane measure for Sts 14 is an estimate from Berge and Goularas [30] as the Sts 14 sacrum is fragmentary and required reconstruction. Sts 65 measures from Claxton et al., [33]. MH2 measures from Kibii et al. [42].

*BSN 49/P27 outlet A-P dimension estimated based on work by Bonmati et al. [44].

Tabun 1^c^ measures from Ponce de León et al. [36] and the measures for Tabun 1^d^ are from Weaver and Hublin [37].

Three published modern human samples are shown to encompass the range of variation in modern humans [^e^[17], ^f^[34]; ^g^[42]]. *Pan troglodytes* measures from Abitbol [16] and Berge and Goularas [30]. All measurements are in millimeters.

To verify and supplement published dimensions for *A*. *sediba* [42], a composite pelvis was reconstructed in AutoDesk Maya^®^ 2015. The pelvic composite consisted of the adult female's (MH2) ilium, pubis, and sacrum and the juvenile male's (MH1) ischium. The *A*. *sediba* fossil specimens are housed in the Evolutionary Studies Institute (ESI) at the University of the Witwatersrand, Johannesburg, South Africa. Three-dimensional surface scans are downloadable at www.morphosource.org. The ESI fossil access committee granted author JMD permission to study the original fossil material discussed in this study. Additional composite pelves were reconstructed with ischia from *A*. *afarensis* (A. L. 288–1), *A*. *africanus* (Sts 14), and *H*. *sapiens* for comparison to the MH1 ischia measures.

### Composite pelvis reconstruction

A first generation cast of the reconstructed MH2 hemipelvis [42] and the left ischium of MH1 (U.W. 88–14) were scanned using a NextEngine^™^ laser HD desktop scanner using the highest-quality settings, 360^o^ scan rotation, macro precision scan exposure and distance, with 16 scan divisions, to create a high-resolution scan. In ScanStudio^™^, an application of NextEngine^™^, each scan was trimmed of excess background noise, exported as an .stl file, and processed using AutoDesk Maya^®^ 2015. The ischium was then attached to the MH2 pelvis to create an *A*. *sediba* composite. To ensure the accuracy of the ischium's placement, it was first aligned with MH2's acetabular notch, using comparative pelvic material from other hominins (A.L. 288–1, Sts 14) and modern humans. Additionally, an arc was created with the three-point circular arc tool in AutoDesk Maya^®^ 2015 to align the lunate surfaces visible in both the MH1 and MH2 acetabula. This allowed us to create an accurate anatomical alignment of both specimens. The acetabula were chosen for alignment because they are approximately the same size in both MH1 and MH2, despite age and sex difference between the individuals [42, 45].

**Fig 1:**
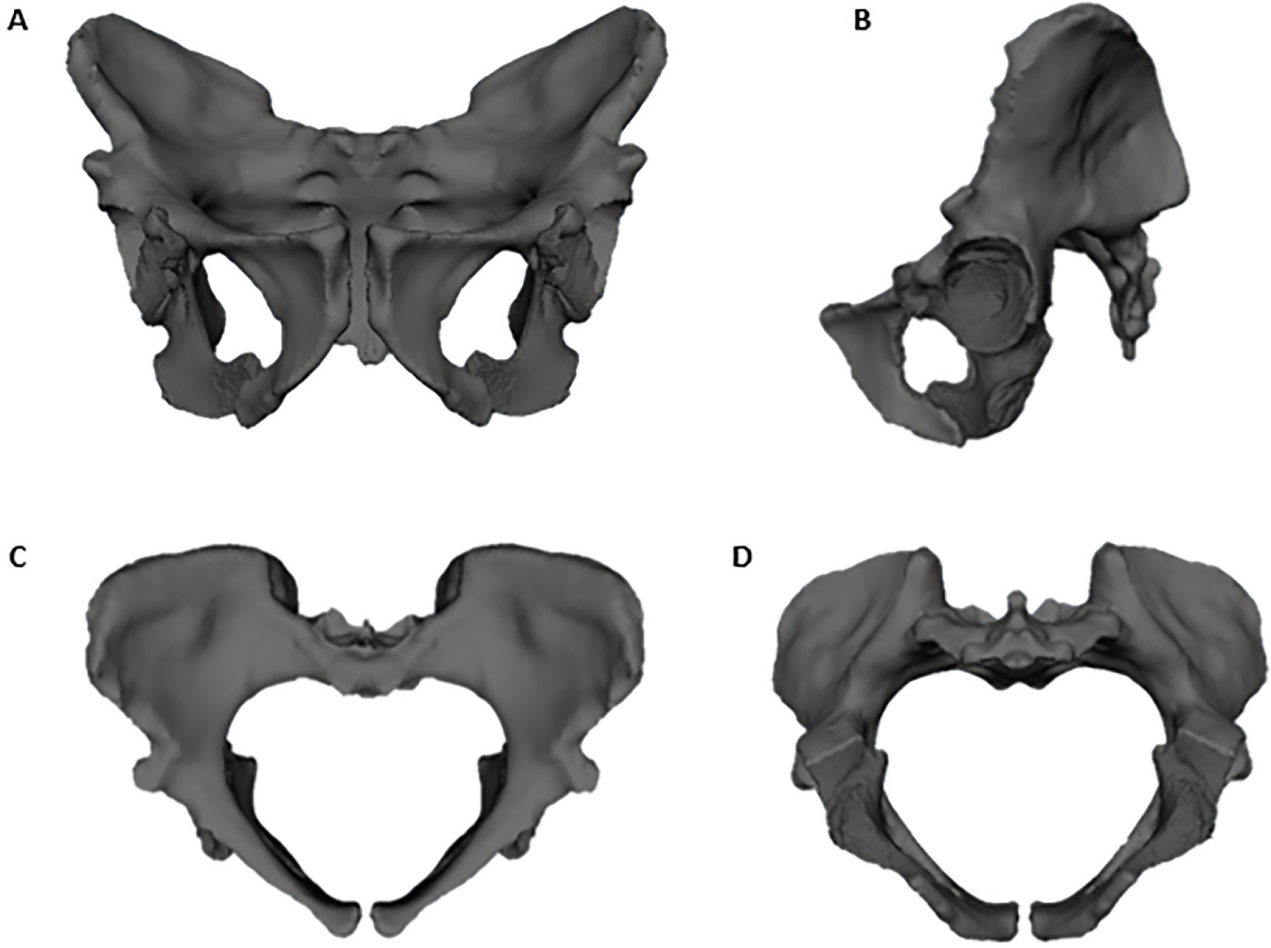
Full pelvis reconstruction of *Australopithecus sediba* using the ischium from MH1. The MH2 hemipelvis was mirror-imaged for the reconstruction. Composite pelvis shown in (A-D) anterior, lateral, superior, and inferior views. Notice that although the MH1 and MH2 acetabulae align, the ischium from MH1 does not cleanly conjoin with the inferior pubis ramus of MH2. This is likely a product of both sex and age differences between MH1 and MH2.

The mirror geometry tool in polygon mode was then used to mirror-image the hemipelvis with the attached MH1 ischium. The two pelvic halves were then aligned at the vertices and merged with the original to produce a single object, representing a full pelvis reconstruction (Fig 1).

To compensate for age and sex differences between the two *A*. *sediba* individuals, additional hominin ischia were aligned to the MH2 reconstruction to produce a range of possible reconstructions. Ischia of A.L. 288–1, Sts 14, and a small-bodied modern human female from the Boston University Anthropology laboratory were scanned and attached to the MH2 pelvis using the same techniques employed above to produce the reconstructions. All ischia were scaled to MH2's acetabular dimensions to control for body size differences and the lunate surfaces were aligned with the three-point circular arc tool. This approach allowed us to assess the effect of using the MH1 male, juvenile ischium in our analysis of birth.

A recent study has called into question the reconstruction of the pelvic anatomy of *A*. *sediba* [46]. Here, we briefly assess these critiques based on observations we have made on the original fossil material. The criticism of the Malapa pelvis reconstruction [46] is centered around six observations: 1.) The reconstruction is based on a single specimen. 2.) The ilia are “grossly” internally rotated in part because there is “no auricular surface” preserved on the lateral sacrum. 3.) The pubis is misaligned because the arcuate line is not continuous. 4.) The superior elevation of the pubic symphysis cannot be assessed because the lower ischial ramus is not preserved. 5.) The symphyseal joint is externally rotated. 6.) Much of the acetabulum is not preserved.

**Fig 2:**
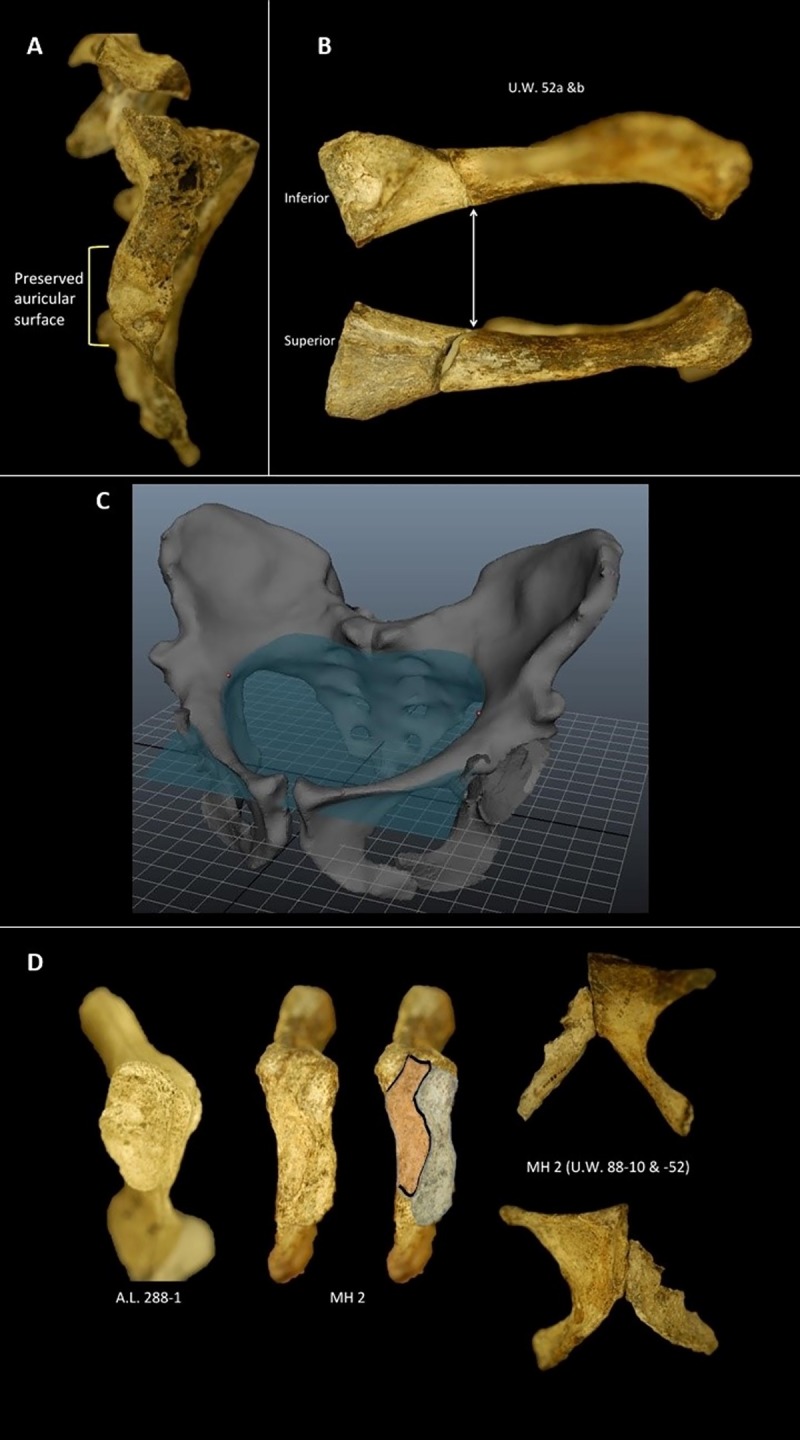
Detailed anatomy of the MH2 pelvis that informed the reconstruction used in this study. A. Preserved auricular surface on the MH2 sacrum. B. Superior pubis showing tight, direct contact between U.W. 88–52 and U.W. 88–136 (previously called U.W. 88-52b). C. Plane fitted to composite pelvis showing the arcuate line is continuous. D. Pubis of MH2 (U.W. 88–52 and U.W. 88–10) articulated. E. Comparison of the pubic symphysis between *A*. *afarensis* (A.L. 288–1) and *A*. *sediba* (MH2). Outlined in gray is the articular surface for the contralateral pubis; outlined in orange is the non-articular portion of the symphysis and presumed insertion for the anterior pubic ligament.

These are important observations and serious critiques that need to be addressed in turn because, if true, they would fundamentally alter the measurements we use to assess the obstetric pelvis of *A*. *sediba*. The critiques of the Malapa reconstruction are addressed in turn below:

1. There is pelvic material for two individuals, not one. MH1, a 12-13-year-old juvenile male [41, 47] preserves four pieces of a pelvis, including two conjoining pieces of a right ilium (U.W. 88–6 and 88–7), a left ilium (U.W. 88–102), and a left ischium (U.W. 88–14) [48]. MH2, an adult female—and the basis of this critique and reevaluation—is better preserved and consists of a nearly complete right ilium (U.W. 88–133), sacrum (U.W. 88–137), right pubis (U.W. 88–52 and 88–136), and left pubis (U.W. 88–10) [48]. There are thus two pelvises (male and female) that can be used to assess pelvic anatomy in *A*. *sediba*—similar to the situation in *A*. *afarensis* (A.L. 288–1 female; KSD-VP-1/1 male). We use both in our reconstruction.
2. It is inaccurate that there is no auricular surface preserved on the lateral aspect of the sacrum. There is damage to the specimen as clearly illustrated in Kibii et al. [42] and Churchill et al. [48]. However, the inferior 11.6 mm of the auricular surface is preserved (Fig 2A). This represents about 1/4th of the total surface. While not ideal, some auricular surface is preserved and anchors the contact between the ilium and the sacrum.
3. The superior pubic ramus was collected in two pieces: U.W. 88-52a and U.W. 88-52b (now called U.W. 88–52 and 88–136). While the reconstruction in Kibii et al. [42] appears to show some infill between these two fragments and thus the potential for misalignment, there is indisputable evidence for direct contact between these fragments. The clean contact between these pieces occurs mostly inferiorly and posteroinferiorly making the contact between these pieces smooth anteriorly and inferiorly (Fig 2B). Furthermore, in our reconstruction, a plane was fit to the arcuate line, assuring that it is continuous (Fig 2C).
4. Lovejoy et al. [46] are correct that the lower ischial ramus is not preserved in MH2, making it difficult to infer superior pubic ramus elevation from the preserved pieces. However, superior deflection of the pubis (or not) would not necessarily alter the obstetric dimensions presented in this study.
5. Kibii et al. [42] reconstructed the right pelvis of MH2 and then mirrored the result to produce their reconstruction. In doing so, the pubis appears externally rotated, perhaps excessively so [46]. Fortunately, one does not have to rely on mirroring the right pelvis. U.W. 88–10 is the left pubis of MH2 and this bone articulates cleanly with U.W. 88–52 (Fig 2D). There is a more posteriorly positioned contact facet on both U.W. 88–52 and 88–10, which is vertically oriented and would be the site of the interpubic disc (Fig 2E). In humans, the disc is typically 1–3 mm thick [49]. Anteriorly, the bones flare outward relative to the vertical and only appear externally rotated because of their orientation relative to the interpubic disc. The anterior pubic ligament would attach here, and in humans is typically 5–12 mm thick [49]. Thus, an externally rotated anterior aspect of the pubic symphysis is normal morphology in MH2 and is not a product of misalignment. The symphyseal joint is therefore not externally rotated, but only appears that way because of the wide anterior spacing—an observation supported by the presence of the left pubis (U.W. 88–10) in MH2.
6. While it is true that the acetabulum of MH2 is not complete, there is enough preserved to assess both the size and morphology, though neither are particularly relevant for this study of obstetrics in *A*. *sediba*.

Thus, many of the criticisms of the MH2 reconstruction are unsupported after a careful examination of the original material. However, as with even the well preserved A.L. 288–1 pelvis [17, 25], there are sure to be alternative versions of the MH2 pelvis (see [50, 51], for example). Whether an alternative pelvic reconstruction will alter the obstetric dimensions used in this paper is currently unclear. Ultimately, the issue for MH2 pelvic reconstruction anatomy is the minimal contact between the U.W. 88–137 sacrum and the U.W. 88–133 ilium; and the tenuous contact between the U.W. 88–133 ilium and the U.W. 88–52 pubis fragment. Fortunately, there is preserved contact at the pubic symphysis, which can be used to anchor the reconstruction anteriorly (Fig 2D). Additionally, a plane has been fit to the arcuate line, demonstrating its continuous nature in this reconstruction (Fig 2C). Nevertheless, MH2 is missing the inferior portion of the ischium, as correctly pointed out by Lovejoy et al. [46].

### Birth canal measurements

Anatomical landmarks referenced in Tague and Lovejoy [17] as markers of the pelvic inlet, midplane, and outlet were identified on the pelvic reconstruction and marked with spherical points in AutoDesk Maya^®^ 2015. The AutoDesk Maya^®^ 2015 distance tool was then used to measure the sagittal and transverse dimensions of the three birth canal planes in the *A*. *sediba* pelvis model. The preserved portion of the MH1 ischium was used for transverse midplane measurement, as the MH2 ischium is fragmentary and lacks the ischial spines [42]. The original description of the pelvis [42] created an estimation of the ischial spine protrusion. We used the preserved portion of the ischium as the marker for our transverse midplane measures because the distance difference between the reconstructed ischial spines and our measurements were less than two millimeters. Each birth canal plane was independently and separately measured by two authors (NML and FR) to ensure replicability. The resulting model produced pelvic dimensions nearly identical to those reported by Kibii et al. [42] (see Tables 1 and 2), though we note that the inlet mediolateral dimensions we calculated are 4.4% smaller than those reported by Kibii et al. [42]. Nevertheless, our composite reconstruction allowed for obstetrically critical measurements (ML midplane and outlet) not reported by Kibii et al. [42]. Measurements of the birth canal planes with the other hominin ischia were used to produce a range of measures for comparison with the use of the male MH1 ischium.

**Table 2:** Composite *A*. *sediba* pelvic measures.

Specimen	Midplane	Outlet	Percent change	
*A*. *sediba* w/MH1 ischium	**96.9**	**104.2**	Midplane	Outlet
*A*. *sediba* w/ A.L. 288–1 ischium	99.4	103.3	2.6%	0.8%
*A*. *sediba* w/ Sts 14 ischium	98.7	107.2	1.9%	2.9%
*A*. *sediba* w/ Modern female ischium	100.4	106.8	3.6%	2.5%

Comparison of computed pelvic midplane and outlet transverse measures when using ischia from: 1. *A*. *sediba* (MH1), 2. *A*. *afarensis* (A.L. 288–1), 3. *A*. *africanus* (Sts 14), 4. *Homo sapiens* (BU 12).

**Fig 3:**
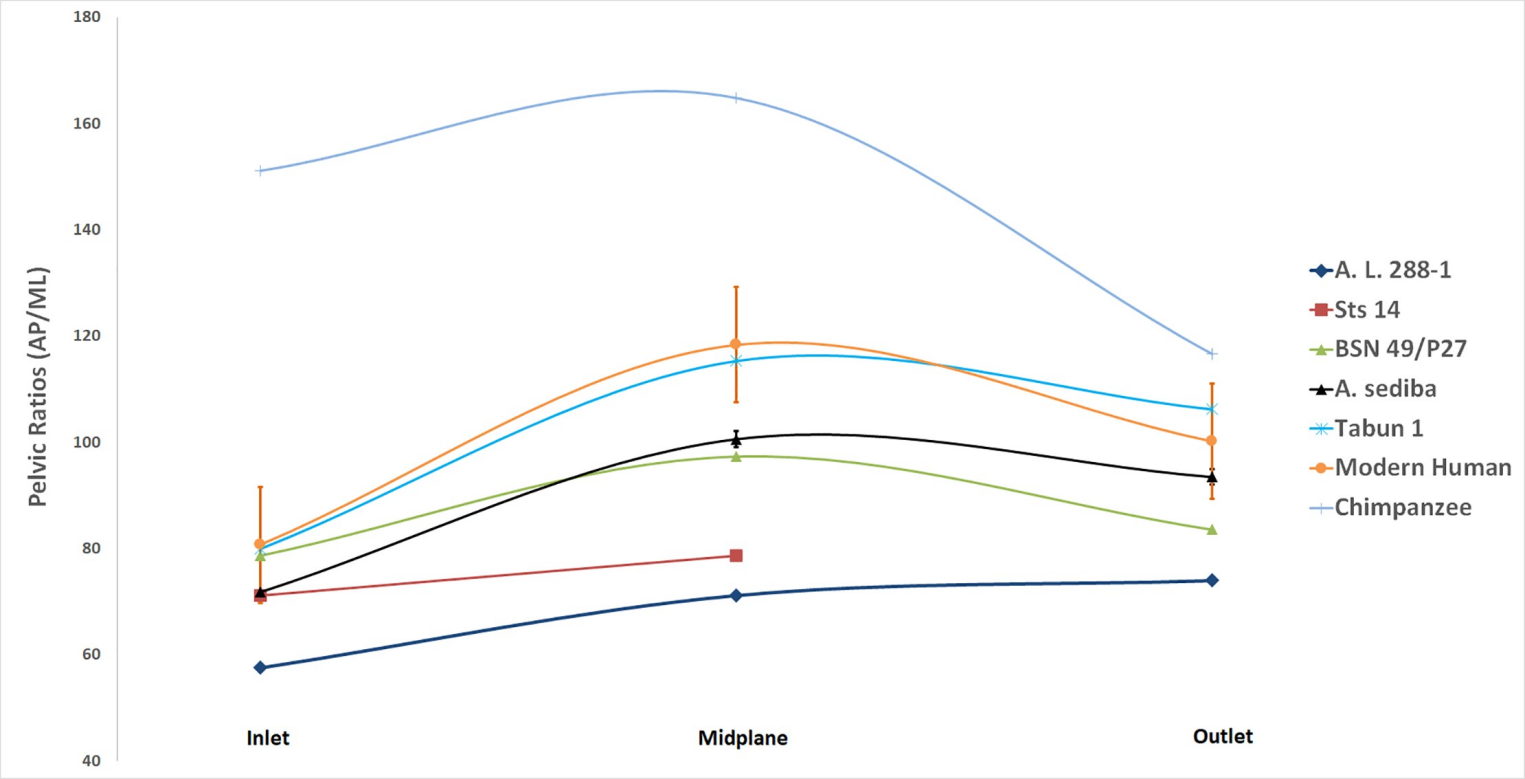
Pelvic indices (AP/ML) for hominin measures reported in Table 3. Notice that the chimpanzee pelvis remains AP elongated throughout the birth canal. Modern humans, in contrast, have a transversely oriented inlet that broadens (AP) at the midplane and becomes rounder at the outlet. Early australopiths (A.L. 288–1 and Sts 14) have transversely wide obstetric dimensions throughout. Notice, however, how similar the birth canal ratios of *A*. *sediba* are to BSN 49/P27, thought by many to belong to fossil *Homo*. The human values incorporate a range based on unpublished data provided by H. Kurki (n = 187, 1 standard deviation) and reported measures in Tague and Lovejoy [17], Simpson et al. [34], and Kibii et al. [42]. The *A*. *sediba* ranges are based on the different ischia (MH1, A.L. 288–1, Sts 14, and small-bodied human) that were used in the different composite pelves reconstructed. Notice that use of these different ischia does not significantly alter the estimated obstetric ratios.

Pelvic shapes were examined using pelvic ratios of the sagittal:transverse dimensions of the pelvic inlet, midplane, and outlet for female modern humans, hominins, and chimpanzees (Fig 3 and Table 4). These ratios allowed us to examine if there were any shape changes between the pelvic planes in *A*. *sediba*. Rotational birth in modern humans occurs, in part, from the pelvic shape change from the transverse inlet to the more sagittal midplane [2, 3, 14, 17]. Evaluation of pelvic shape change in *A*. *sediba* is therefore important for making a complete analysis of the birth mechanism in *A*. *sediba*.

### Neonatal cranial dimensions and volume

Neonatal cranial dimensions (length, breadth, and height) were calculated utilizing a catarrhine regression model [52] to estimate a neonate brain volume, from which we calculated the fetal skull dimensions. The cranial dimensions from the regression model were then used to construct an ellipsoid shape in AutoDesk Maya^®^ 2015 to provide visual reference of the neonate's hypothetical progression through the birth canal.

To calculate the neonatal cranial length, breadth, and height, a regression-based estimate of neonatal cranial capacity was applied to the published brain size of MH1 [41, 52]. *Australopithecus sediba* cranial capacity (420cc) is known for only a single juvenile (MH1) aged 12–13 years old at time of death [41, 47] but is likely no different than an adult given that both chimpanzees and humans attain adult brain mass by the age of six-to-seven years old [53, 54]. The neonatal cranial capacity of an *A*. *sediba* was estimated using the least squares equation (from [52]):

log(neonatalbrainmass)=0.77xlog(adultbrainmass)+0.19

The neonatal cranial volume from the regression formula was then used to calculate the neonatal cranial length, breadth, and height using an ellipsoid volume formula ($\frac{4}{3}\pi \times r_{1}\times r_{2}\times r_{3}$) [33]. Cranial biparietal breadth (BP), fronto-occipital length (FO) and height (H) were substituted into the ellipsoid formula, ($\frac{4}{3}\pi \times \left(\frac{BP}{2}\right)\times \left(\frac{FO}{2}\right)\times (\frac{H}{2})$). Known human neonatal cranial ratios [34] were calculated using the biparietal breadth to then calculate the fronto-occipital length (FO) and height (H): ($\frac{4}{3}\pi \times \left(\frac{BP}{2}\right)\times 1.22\left(\frac{BP}{2}\right)\times 0.65(\frac{BP}{2})$) (see Table 4).

An ellipsoid shape was then created to simulate the neonatal *A*. *sediba*'s head using the polygon primitives tool in AutoDesk Maya^®^ 2015. The ellipsoid dimensions (length, breadth, and height) were measured to the dimensions specified from the above calculations. The digital head was then inserted into the composite pelvis with the MH1 ischium. Birth mechanisms were simulated by aligning the ellipsoid sagittally, transversely, and obliquely at all birth canal planes (inlet, midplane, outlet). A motion path arc allowed for an animation of the full "birth" process of the ellipsoid through the pelvis and to provide a visual representation of any obstruction.

### Shoulder constraints

Because ephalopelvic constraint is not the only potential obstruction faced during birth, neonatal biacromial breadth was estimated to test if shoulder dystocia was a risk during birth in *A*. *sediba*. There is no neonatal *A*. *sediba* clavicle known; however, there is an adult clavicle, which at 107.5 mm long is considerably shorter than the average clavicular length in an adult chimpanzee, gorilla, or human [55]. In primates, adult clavicle length is a strong predictor of neonatal biacromial breadth [15]. Therefore, we were able to estimate a neonatal biacromial breadth from the adult clavicle using the least squares regression equation: y = 0.68x + 0.49 where y = log(neonatal biacromial breadth) and x = log(adult clavicular length). The resulting estimated neonatal biacromial breadth for *A*. *sediba* is 74.3 mm.

## Results

### Composite pelvis reconstruction with MH1 ischium

The composite *Australopithecus sediba* pelvic reconstruction is shown in Fig 1. The MH2 portions of the pelvis illustrate the mix of derived and primitive features described in the original reconstruction by Kibii et al. [42] and again by Churchill et al. [47]. The MH2 pelvis shares with other australopiths a relatively long pubis, small sacral joints, and a relatively wide biacetabular diameter [42]. The MH2 pelvis shares with *Homo* an AP expansion at the pelvic inlet [42] and at the midplane (this study). The addition of the MH1 ischium allows for a more complete analysis of the birth canal pelvic planes, as the transverse dimension of the midplane is important in determining whether the neonatal head rotates through the birth canal, as it does in modern humans. The ischiopubic rami of the two specimens (MH1 and MH2) are not perfectly aligned, probably reflecting a sex difference, but the acetabula are the same size and conjoin without any scaling of either specimen. This reconstruction, and the obstetric results derived from it, should be reassessed should the MH2 ischium, or a more complete adult female *A*. *sediba* pelvis, be found.

### Composite pelvic reconstruction with other hominin ischia

The ischia of *A*. *afarensis*, *A*. *africanus*, and *H*. *sapiens* also show incongruence with the inferior rami of the MH2 model, in this instance, probably reflecting species differences (Table 2). Midplane and outlet transverse dimensions from these female hominin specimens illustrate that there is less than a 4% difference than when using the MH1 specimen (see Table 2). Only in one measure, the outlet transverse dimension with the *A*. *afarensis* ischia, is the measure smaller (<1mm) than when using the MH1 ischium.

### Obstetric measurements

The sagittal and transverse measurements of the pelvic inlet, midplane, and outlet are reported in Table 3. The indices of these planes are shown in Table 4 and graphed in Fig 3 to illustrate the pelvic shapes of different hominins.

**Table 3:** Estimated obstetric plane measures for *A*. *sediba*.

Specimen	Inlet–AP	Inlet–ML	Midplane–AP	Midplane—ML	Outlet–AP	Outlet–ML
*A*. *sediba* w/MH1 ischium	80.8	112.4	97.5	96.9	97.4	104.2
*A*. *sediba* w/ A.L. 288–1 ischium	80.8	112.4	97.5	99.4	97.4	103.3
*A*. *sediba* w/ Sts 14 ischium	80.8	112.4	97.5	98.7	97.4	107.2
*A*. *sediba* w/ Modern female ischium	80.8	112.4	97.5	100.4	97.4	106.8

MH2^2^ estimated measurements taken for this study from the composite reconstruction of the MH1 and MH2 pelvic remains with ranges from other hominins (A.L. 288–1, Sts14, and *H*. *sapiens*).

**Table 4:** Ratio of pelvic planes (AP/ML x 100) based on measures reported in Tables 1 and 3.

Specimen	Inlet (AP/ML)	Midplane (AP/ML)	Outlet (AP/ML)
A. L. 288-1^a^	57.6	71.2	74.0
A. L. 288-1^b^	64.3	129.7	108.3
Sts 14	71.2	78.7	NA
BSN 49/P27	78.7	97.4	83.6
*A*. *sediba*	71.9	97.1–100.6	91.2–94.3
Tabun 1^c^	79.9	115.3	106.2
Tabun 1^d^	79.4	NA	70.5
Modern Human^e^	73.2–83.0	116–127	96.7
Modern Human^f^	86.6	120.7	103.7
Chimpanzee	151.1	164.8	116.6

A.L. 288-1^a^ measures from Tague and Lovejoy [17].

A.L. 288-1^b^ ratios are the average of two reconstructions from Häusler and Schmid [25]. The range reported for *A*. *sediba*’s midplane and outlet indices reflect the estimated measures derived from utilization of the other hominin ischia.

Tabun 1^c^ average measurements used from range reported by Ponce de León et al. [36].

Tabun 1^d^ measures from Weaver and Hublin [37].

The modern human^e^ range encompasses the average values reported in Tague and Lovejoy [17], Simpson et al. [34], and Kibii et al. [42].

Modern human^f^ is the average of data (unpublished) provided to the authors by H. Kurki (n = 187).

*Australopithecus sediba* exhibits a pelvic shape unlike the consistently platypelloid pelvis of *A*. *afarensis* (A.L. 288–1) and *A*. *africanus* (Sts 14) (Fig 3). At the pelvic inlet, the pelvic ratio of *A*. *sediba* are nearly identical to *A*. *africanus* (Sts 14, Sts 65), midway between *Homo* and *A*. *afarensis*. However, the pelvic midplane in *A*. *sediba* exhibits a *Homo*-like anterior-posterior expansion, not seen in the other australopiths. The outlet is more rounded, with an index similar to modern humans.

### Fetal cranial volume and dimensions

Using the LSQ regression equation [51], *Australopithecus sediba* most likely birthed infants with a brain of 162.1 cc (range based on 95% CI of regression equation: 145.8 cc-180.4) (Table 5). Given the values from the LSQ regression equation, the *A*. *sediba* neonate would have a cranium with a biparietal breadth of ~73 mm and a frontal-occipital length of ~89 mm, only slightly larger than a neonatal chimpanzee cranium.

**Table 5:** Estimated neonatal cranial dimensions for *A*. *sediba*. Regression-based estimate of neonatal cranial capacity using the published brain size of MH1 from methods in [52].

Volume (cm^3^)	Biparietal Breadth (mm)	Fronto-occipital length (mm)	Cranial height (mm)
162.1	73.1	89.2	47.5

### Digital neonatal cranium

**Fig 4:**
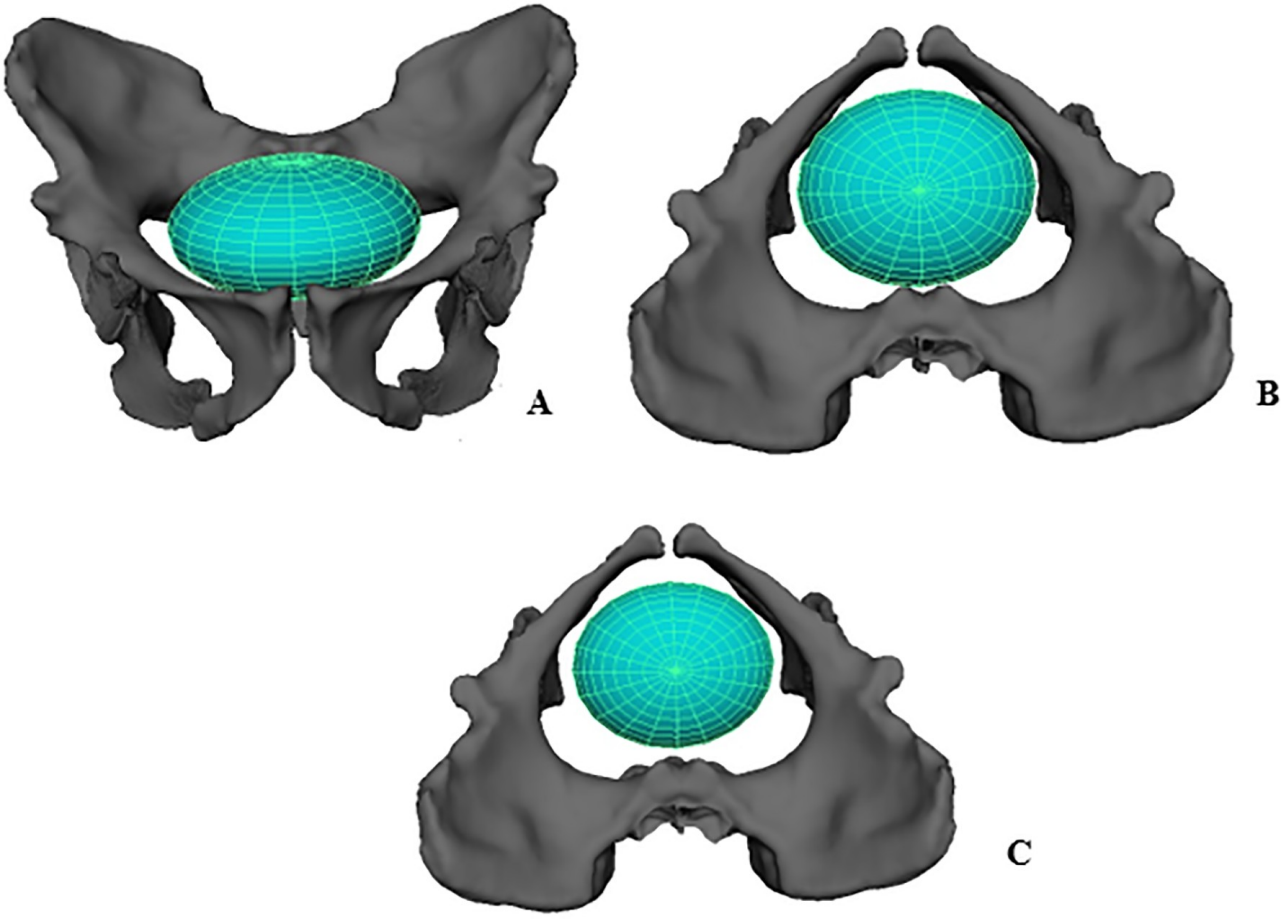
Ellipse representing a neonatal *A*. *sediba* head at the pelvic. **A.** inlet, frontal view **B.** inlet, superior view **C.** midplane, superior view. Reconstructed pelvis is shown with the MH1 ischium. Notice that the modeled *A*. *sediba* neonatal cranium can descend into the midplane without bony constraints, unlike the condition typically found in modern humans.

A digital cranium for an *A*. *sediba* neonate was simulated with an ellipsoid shape in AutoDesk Maya^®^ 2015 (Fig 4). As expected from comparing the cranial dimensions to the pelvic inlet measures, the fronto-occipital length of the neonate cranium (89.2 mm) was too long to pass sagittally through the AP diameter of the inlet (80.8 mm). Aligning the cranium transversely in the inlet (112.4mm), the cranium could pass through without constraint, similar to birth reconstructions in other hominins and humans [15–17, 23, 26, 30].

The midplane of the *A*. *sediba* pelvis shows a shift in pelvic dimensions with the transverse dimension becoming constrained due to the ischial spines. However, a transverse orientation of the fetal head is possible even with a minimum transverse midplane dimension of 96.9mm using the MH1 ischium (Table 5, Table in S1 Table). Therefore, the fetal head may have remained transversely oriented at the midplane.

Comparison of the midplane shape to other hominins represented in Fig 3 shows that the AP elongation in *A*. *sediba* is more similar to the shift seen in genus *Homo* than other australopiths (*A*. *afarensis* and *A*. *africanus*). This result holds when the MH1 ischia is replaced by female hominins of *A*. *afarensis*, *A*. *africanus*, and *H*. *sapiens* (Fig 3).

The outlet of the pelvis shows a slight transverse expansion with a continued constrained AP dimension and the simulated cranium was able to pass through transversely. However, again the outlet shape was more consistent with members of the genus *Homo* than the other australopith (A.L. 288–1), even substituting the other hominin ischia (Fig 3).

### Neonatal biacromial breadth

The estimated biacromial breadth for an *A*. *sediba* neonate would be 74.3mm. When the neonatal shoulders are at the pelvic inlet, the fetal head would be at approximately the midplane and in a transverse orientation. The fetal shoulder breadth is small enough enter the pelvic inlet sagittally, maintaining a perpendicular orientation to the fetal head. The pelvic inlet sagittal dimension is the most constrained dimension of the pelvis, resulting in the shoulders not needing to change orientation throughout the rest of the pelvic planes.

## Discussion

The discovery of female hominin pelvic remains helps to inform how the complex mechanism of human birth evolved. Chimpanzees, our closest living relatives, have relatively easy births. Small neonatal head size combined with a more spacious and uniformly shaped birth canal makes birth a rapid and relatively easy event that does not benefit from birth assistance. Humans, however, pair an enlarged neonatal cranial capacity with a birth canal that changes dimensions, resulting in fetal rotation in the birth canal. However, when rotational birth arose in human evolution remains unknown. Comparison of the birth mechanisms in fossil hominins has yielded varied results [15, 17, 23, 25, 30, 34, 37]. The composite pelvis achieved in this study allowed us to evaluate how birth may have occurred in *A*. *sediba*, a species that possesses a small cranial capacity, yet a more *Homo*-like pelvis with AP expansion of the birth canal. If *A*. *sediba* possessed obstetric challenges beyond that found in other australopiths, then perhaps these changes are related to obstetrics. This reconstruction of birth permits a test of the hypothesis that pelvic morphology in *A*. *sediba* was adapted for locomotion, rather than obstetrics [42].

The cranial dimensions and capacity for an *A*. *sediba* neonate were estimated using a regression-based analysis. With the LSQ regression equation, an estimated *A*. *sediba* neonatal brain volume of 162.1cc was predicted. The calculated cranial dimensions of biparietal breadth, fronto-occipital length, and brain height were then compared to the pelvic inlet, midplane, and outlet dimensions of the MH2 reconstruction to examine if rotational birth in *A*. *sediba* occurred.

Our results indicate that a neonate of *A*. *sediba* would have had a transverse entry into the pelvic inlet, as has been suggested for other species of *Australopithecus* [17, 23, 26, 33]. The anterior-posterior dimension of the *A*. *sediba* pelvic inlet is too constrained to allow a frontal-occipital passage of a neonatal cranium, making a transverse or oblique entry the most likely option.

After the transverse descent through the pelvic inlet, the fetal head would have room to continue transversely through the pelvic midplane. The MH1 ischium provides the most constricted dimensions due to the age and sex of the specimen. Although MH1 is a male specimen and therefore its use in an obstetrics analysis is unconventional, it is the only ischium assigned to *A*. *sediba*. The utilization of the MH1 ischium provides a minimum estimation of obstetric dimensions in MH2. However, even with these minimum dimensions (96.9mm), the fetal head length (89.2mm) would occupy 92.1% of the transverse dimension of the midplane, providing sufficient space for the fetal head to pass in a transverse orientation. If the fetal head and shoulder breadth can fit through these dimensions, it can be assumed that the neonate would also fit through the expanded dimensions a female ischium would afford. For midplane rotation to be necessary for the composite reconstructions in this study, the *A*. *sediba* neonatal brain size would need to increase 28.2–42.6% beyond the estimated 162.1cc. Back calculating from the LSQ regression equation, such an increase would predict an adult *A*. *sediba* brain volume of 572-664cc. This adult brain volume considerably exceeds the one known *A*. *sediba* brain volume (420cc) and is greater than any known australopith. As rotational movement by the fetus could result in a more difficult and complex birth, it is possible that fetal descent remained transversely oriented at the midplane. The pelvic outlet in *A*. *sediba* also does not exhibit bony obstruction relative to the neonatal cranial dimensions. The neonate would have had room to continue transversely, or obliquely out of the pelvic outlet. The transverse dimension of the outlet expands slightly to a length 7% longer than the estimated *A*. *sediba* neonatal cranium, not accounting for soft tissues. Any flexion of the neonate’s head would further decrease the diameter of the fetal head during the fetal exit of the birth canal under the pubic symphysis [3].

We have shown that this mechanism of birth—one predicted for A.L. 288–1 [17]—would increase the risk of shoulder dystocia in *A*. *afarensis* and is thus problematic [15]. In A.L. 288–1, the estimated neonatal shoulder breadth greatly exceeds the obstetric dimensions [15]. However, shoulder breadth would not have contributed to obstetric constraints with any pelvic dimensions in *A*. *sediba*. Even at their most constrained location (the AP pelvic inlet [80.8mm]), shoulder breadth (predicted to be 73.4 mm wide) could still pass unimpeded and may have been further reduced by cranially elevating the clavicles, “shrugging” to enter the birth canal [14]. The other pelvic planes had ample room for shoulder passage without the risk of dystocia.

A similar birth mechanism has been suggested for other australopiths where the baby enters the pelvic inlet aligned transversely, but requires no further rotations [16, 17, 23, 25, 26] (but see [15, 30]) and is perhaps unsurprising given some of the primitive anatomies of *A*. *sediba* [42]. With the estimated neonatal cranial and maternal pelvic dimensions utilized in this study, non-rotational birth is possible in *A*. *sediba*. Nevertheless, the interspecific differences in fossil hominin pelvic morphology and fetal dimensions show that there is not a linear, gradual change from an “easy” birth to a “difficult” birth. Instead, the morphology of each specimen exhibits its own set of obstetric challenges.

Hominin pelvic morphology is thought to be influenced by both locomotion [42] and obstetrics [34, 56]. The increase in encephalization throughout the hominin lineage has previously been thought to be the driving factor in expanding the AP dimensions of the pelvis (i.e. [34]). However, *A*. *sediba* possesses an AP expanded, *Homo*-like pelvis, with little evidence for obstetric constraints. This finding suggests that at least in *A*. *sediba*, the morphology of the pelvis was probably shaped by locomotion factors rather than solely obstetrics.

### Rotational birth

The more gynecoid pelvis of early *Homo* may have been a result of obstetric requirements [34] and may have resulted in rotational birth [56]. However, *A*. *sediba* also possesses AP expansion in the pelvic midplane and raises the possibility of rotational birth in this taxon. To accommodate this interpretation, we describe fetal descent following determinations of Joulin’s Law which states that the neonate would rotate to coincide with the maximum dimension of the bony anatomy [18]. In the *A*. *sediba* pelvis, the maximum bony dimensions are not always in the transverse dimensions (Table 3). Therefore, following the assumption that the neonate would align to these maximum dimensions, a non-rotational birth pattern may not be the default for *A*. *sediba*. Following passage through the ML broad pelvic inlet, the midplane of *A*. *sediba* shows an anterior-posterior expansion to an even greater degree than other australopiths, making it more *Homo*-like. While the *A*. *sediba* neonate could pass through this plane transversely, there would be more space if it rotated and aligned the fronto-occipital length of the skull with the wider anteroposterior dimension of the maternal pelvis.

*Australopithecus* midplane rotation has been proposed for *A*. *africanus* (i.e. Sts 14) by Berge et al. [23] and Berge and Goularas [30] who cite not only the increased AP expansion of the bony anatomy, but also uterine forces that will direct the neonate to rotate in this plane. As exhibited in Fig 3, the pelvic shape changes more dramatically from the inlet to the midplane in *A*. *sediba* than either the *A*. *africanus* or even *H*. *erectus* specimens. Instead, the shape change is more consistent with what is seen in Neandertals (Tabun 1) and modern humans. Additionally, this shape change is notable since *A*. *africanus* and *A*. *sediba* start at similar inlet indices.

The final component to a difficult modern human birth is the fact that rigid shoulders cannot pass through the changing shape of the birth canal without some rotation [2, 3, 14]. Using the estimated neonatal shoulder breadth, the shoulders would not contribute to obstetrical obstruction in *A*. *sediba* in either a transverse or rotational birth scenario.

We caution that our results are contingent on our reconstruction of the MH2 pelvis. As mentioned previously, the tenuous contact between the ilium and the sacrum in addition to the use of the MH1 ischium introduce the potential for error. We therefore anticipate that the findings presented here will be revisited should new pelvic fossils of *A*. *sediba* be found or should another team reconstruct the available material in a manner morphologically distinct from that presented here and elsewhere [42, 47].

## Conclusion

Reconstructing the pelvis of a female *Australopithecus sediba* (MH2) provides an assessment of the birth process in this Early Pleistocene hominin species. At the pelvic inlet, the neonatal head aligned with the maximum dimension of the pelvic inlet to enter the birth canal transversely. Lack of bony impingement into the birth canal, combined with a small neonatal head size would not necessitate further rotation of the fetus as it descended through the canal, though AP expansion of the maternal pelvis still indicates that rotational birth may have occurred. It is possible, even, that there was considerable variation in the birth mechanism in early australopiths, with varying amounts of neonatal rotation. Interestingly, the shape of the obstetric planes in *A*. *sediba* align more closely with the genus *Homo* than with the other australopiths. These findings imply that the anteroposterior expansion of the birth canal can occur without neonatal brain expansion in early hominins.

## Supporting information

S1 Table This table shows all the data used for this study. BP: neonatal cranium biparietal breadth FO: frontal-occipital length of neonate cranium. AP: anterior-posterior dimension of maternal pelvis or composite pelvis ML: transverse dimension of maternal pelvis or composite pelvis.
(DOCX)
