# Supplementary material for: Reconstructing birth in Australopithecus sediba
Source: PLoS One. 2019 Sep 18;14(9):e0221871. doi: 10.1371/journal.pone.0221871 (PMC6750590; doi:10.1371/journal.pone.0221871)
Supplement: S1 Table — BP: neonatal cranium biparietal breadth FO: frontal-occipital length of neonate cranium. AP: anterior-posterior dimension of maternal pelvis or composite pelvis ML: transverse dimension of maternal pelvis or composite pelvis. (DOCX) [file pone.0221871.s001.docx]

**Supplementary**

**Table S1: This table shows all the data used for this study.** BP: neonatal cranium biparietal breadth FO: frontal-occipital length of neonate cranium. AP: anterior-posterior dimension of maternal pelvis or composite pelvis ML: transverse dimension of maternal pelvis or composite pelvis.

| Pelvis dimension measured (mm) | *A. sediba* | 5% | 8% |
| --- | --- | --- | --- |
| AP inlet | 80.8 mm |  |  |
| AP midplane | 97.5 mm |  |  |
| AP outlet | 97.4 mm |  |  |
| ML inlet | 112.4mm |  |  |
| ML midplane | 96.9 mm |  |  |
| ML outlet | 104.2 mm |  |  |
| Brain ht:  60%  65%  70%  [34] |  | 46.1  49.9  53.8 | 44.6  48.3  52.1 |
| Ellipsoid equation  $\frac{4}{3}\pi\times r_{1}\times r_{2}\times r_{3}$ |  | 188.0cc | 170.7cc |
| LSQ Regression Equation [50] |  | 166.9cc |  |
| Back calculation from 162.1cc through ellipsoid formula |  | BP: 73.1  FO: 89.2  Ht: 47.5 |  |
| BP% of pelvic inlet |  | 91.3% |  |
| FO% of pelvic inlet |  | 80.1% |  |
| Human neonatal biacromial breadth [14] | 109.3 mm |  |  |
| Chimpanzee Neonatal Biacromial breadth | 84.3 mm |  |  |
| *A. sediba* neonatal biacromial breadth | 74.3mm | 92.9% AP inlet |  |
